# Characterization and assessment of the sensitivity and resistance of a newly established human gastrointestinal stromal tumour xenograft model to treatment with tyrosine kinase inhibitors

**DOI:** 10.1186/2045-3329-4-10

**Published:** 2014-08-10

**Authors:** Thomas Van Looy, Yemarshet Kelemework Gebreyohannes, Agnieszka Wozniak, Jasmien Cornillie, Jasmien Wellens, Haifu Li, Ulla Vanleeuw, Giuseppe Floris, Maria Debiec-Rychter, Raf Sciot, Patrick Schöffski

**Affiliations:** 1 Laboratory of Experimental Oncology, Department of Oncology, KU Leuven, Herestraat 49, Leuven, Belgium; 2 Department of General Medical Oncology, Leuven Cancer Institute, University Hospitals Leuven, Herestraat 49, Leuven, Belgium; 3 Department of Pathology, KU Leuven and University Hospitals Leuven, Herestraat 49, Leuven, Belgium; 4 Department of Human Genetics, KU Leuven and University Hospitals Leuven, Herestraat 49, Leuven, Belgium

**Keywords:** Xenograft, Gastrointestinal stromal tumour, Resistance, Tyrosine kinase inhibitor, Imatinib, Sunitinib, Regorafenib

## Abstract

**Background:**

Acquired resistance to tyrosine kinase inhibitors (TKIs) in gastrointestinal stromal tumours (GISTs) is most commonly caused by secondary *KIT* or *PDGFRA* mutations. In this study we characterize a newly established GIST xenograft model, UZLX-GIST9, and evaluate the *in vivo* response of the model to standard TKIs (imatinib, sunitinib, and regorafenib).

**Methods:**

Tumour fragments from a metastatic lesion of a GIST patient clinically progressing after treatment with imatinib, sunitinib and regorafenib were engrafted in a nude, immunodeficient mouse. Upon sequential passaging from mouse to mouse, tumour fragments were collected for histopathological and molecular characterization. The sensitivity of the model to treatment with TKIs was evaluated in 28 mice [passage 2 (n = 8), passage 4 (n = 20), 41 tumours]. Mice were grouped as follows: control (untreated), imatinib (50 mg/kg/BID), imatinib (100 mg/kg/BID), sunitinib (40 mg/kg/QD), and regorafenib (30 mg/kg/QD). After three weeks of oral treatment, tumours were collected for subsequent analysis. The efficacy of treatment was assessed by tumour volume, histopathology and Western immunoblotting.

**Results:**

UZLX-GIST9 maintains the same typical morphological features and immunohistochemical characteristics as the original patient biopsy and expresses CD117 and DOG1. The *KIT* mutational profile (p.P577del + W557LfsX5+ D820G) remains the same as the original tissue sample originating from an intraspinal metastatic site. Three week treatment with different TKIs showed that the model is resistant to imatinib. Sunitinib induces tumour growth delay and regorafenib reduces the tumour burden by 30% as compared to control animals. While none of the TKIs had a significant effect on cell proliferation or cell survival, a remarkable increase of necrosis and significant reduction of microvessel density was observed under sunitinib and regorafenib. Western immunoblotting showed a mild reduction in KIT and AKT activation only in regorafenib treated tumours.

**Conclusions:**

We established a novel human GIST xenograft, UZLX-GIST9, harbouring *KIT* exon 11 and 17 mutations and maintaining the pheno-and genotype of the original tumour. UZLX-GIST9 shows different levels of response to standard TKIs. This model will help to study TKI resistance and to explore novel treatment approaches for patients with TKI-resistant GIST.

## Background

Gastrointestinal stromal tumour (GIST) is the most common sarcoma of the digestive tract and in some European regions the most frequently occurring mesenchymal malignancy, with an estimated incidence of 12–14.5 per million people [1-3]. To date, surgery remains the cornerstone in the clinical management of primary resectable GISTs [4]. However, surgery is not feasible in all patients because of tumour site, tumour size or due to metastatic spread [5]. Among patients qualifying for primary surgery, 40-50% develops recurrence or metastases during follow-up. In the vast majority of GISTs, activating mutations in either the *KIT* or the *PDGFRA* (platelet derived growth factor receptor alpha) gene are the main oncogenic drivers [6,7]. These genes encode for receptor tyrosine kinases (RTKs), and activating mutations in the according genes can result in constitutive activation of intracellular signalling pathways leading to enhanced cell proliferation and -survival. The clinical significance of this observation is demonstrated by the exceptional anti-tumour activity of tyrosine kinase inhibitors (TKIs) in patients with advanced GISTs. Currently, imatinib is the standard first-line treatment for metastatic and unresectable GISTs and is very well tolerated in the vast majority of patients [8]. Imatinib is a multi-targeted TKI inhibiting ABL, KIT and PDGFRA/B [9]. Unfortunately, with time patients with imatinib-sensitive disease inevitably develop resistance to this agent. Sunitinib is the approved second-line therapy for patients intolerant or no longer responding to imatinib [10]. Sunitinib is an oral multi-targeted TKI with activity against RTKs like KIT, VEGFR1/2/3 (vascular endothelial growth factor receptor) and PDGFRA/B, and was shown to increase progression-free survival as compared to placebo in imatinib-refractory patients in a clinical phase 3 trial [11-13]. Nevertheless, with time the majority of patients will also develop progressive disease under treatment with sunitinib [14]. Recently, regorafenib has been approved by the United States Food and Drug Administration (FDA) as third-line treatment for patients with advanced GIST after failure of both described TKIs. Regorafenib is an orally bioavailable multi-targeted TKI with known activity against KIT, RET (rearranged during transfection), VEGFR1/2/3, PDGFRβ, FGFR (fibroblast growth factor receptor) [15]. In a very recent randomized phase 3 clinical trial regorafenib yielded a significantly better median progression-free survival than placebo (4.8 *versus* 0.9 months), in this setting [16]. When analysing the available phase 3 evidence for all three established agents, it is obvious that the time to progression decreases progressively with every line of TKI treatment. It seems unlikely that the development of further KIT- or PDGFRA-targeted TKIs will circumvent the occurrence of heterogeneous TKI resistance in GISTs [17]. In the majority of resistant GISTs, TKI resistance is mediated by the occurrence of secondary mutations in *KIT* or *PDGFRA*[17]. Some additional resistance mechanisms have been described, like genomic amplification of *KIT* or *PDGFRA* genes or a switch of KIT dependency to other RTKs (e.g., AXL) [18]. Hence, the development of novel GIST research models characterized by different sensitivity to standard treatments is very important for the testing of novel treatment approaches. At present, there are no GIST xenograft models described in the literature that have shown resistance to multiple TKIs. For this reason we are trying to develop novel GIST xenograft models reflecting the resistance pattern observed in the clinic. Our new model UZLX-GIST9 is derived from a patient clinically and radiologically progressing after treatment with imatinib, sunitinib, and regorafenib. In the current study, we have characterized this model and tested its sensitivity *in vivo* to standard treatments.

## Methods

### Patient history

A 66-year old female patient was diagnosed with a mass protruding into the gastric lumen and with synchronous omental metastases. She underwent a total gastrectomy and the pathological examination revealed a GIST with CD117 (KIT)-immunopositivity. Mutational analysis showed a *KIT* exon 11 mutation (p.P577del). The patient was referred to our hospital 18 months later because of progressive disease and started the treatment with imatinib 400 mg daily. After 14 months of therapy, disease progression was observed and the imatinib dose was escalated to 800 mg daily, which also resulted in progressive disease (new thoracic metastases). Then the second-line therapy with sunitinib 50 mg daily was started using the labelled regimen, achieving the stable disease for 3.5 years. Upon the next disease progression, nilotinib 400 mg p.o. twice daily was started under a compassionate use protocol, until disease progression three months later. At that point, imatinib rechallenge was introduced (400 mg p.o. daily), but stopped three months later because of disease progression. The patient was then included in the multicenter, double-blind, randomized phase III trial comparing placebo *versus* regorafenib given at a dose of 160 mg daily, three weeks on treatment followed by a one-week rest period [16]. Upon disease progression three months later, the study treatment was unblinded and the patient was crossed over from placebo to regorafenib, with the disease stabilization for one year. At that point, she was admitted to the hospital with acute muscle weakness and diminished sensitivity in the lower extremities. Magnetic resonance imaging revealed spinal cord compression through direct intraspinal tumour extension. She underwent urgent surgical decompression, during which tumour fragments collected was used for the establishment of the xenograft model. Pathological examination the diagnosis of metastatic GIST and the mutational analysis revealed mutations in *KIT* exon 11 (p.P577del; W557LfsX5) and exon 17 (p.D820G). The patient eventually died of disease 4 months later, 10 years after the initial diagnosis.

### Establishment of the UZLX-GIST9 xenograft model

In April 2012, tumour tissue from an intraspinal GIST metastatic site was collected in culture medium immediately after surgery. The xenografting of patient-derived mesenchymal tumour material is approved by the Medical Ethics Committee, University Hospitals Leuven (S53483). The tumour specimen was cut in small fragments of 50-100 mm^3^ and immediately implanted subcutaneously on both flanks of 1 nude NMRI nude mice (Janvier). After successful engraftment and growth of the tumour, the xenograft model was called UZLX-GIST9. The xenograft could be maintained by serial passaging.

### Molecular characterization

DNA isolation from frozen fragments originating from UZLX-GIST9 ex-mouse tumours was performed using QIAamp DNA Mini Kit (Qiagen). *KIT* mutational analysis was done as previously described, using [ENSG00000157404] as the reference sequence [19].

### Drugs and reagents

Imatinib, sunitinib, and regorafenib were purchased from Sequoia Research Products Ltd., and were dissolved in sterile water, citric buffer (pH 3.5), and 125 mM polyethylene glycol 400/methane sulphonic acid (80/20) respectively. All solutions were stored at 4°C and protected from light.

The following primary antibodies were used for Western immunoblotting and immunohistochemistry (IHC): phospho-Y719 KIT (pKIT Y719), phospho-S473 AKT (pAKT), AKT, alpha-tubulin, phospho-T202/Y204 MAPK (pMAPK), p42/44 MAPK, phospho-S65 4E-BP1 (p4E-BP1), 4E-BP1, phospho-Histone H3 (pHH3) (Cell Signaling Technologies); p-Y703 KIT (pKIT Y703; Invitrogen); KIT (DAKO); beta-actin (Sigma-Aldrich); cleaved PARP (Cl-PARP; Abcam); Ki-67 (Thermoscientific); and DOG1 (discovered on GIST-1; Novocastra). For Western immunoblotting horse radish peroxidase (HRP)-conjugated antibodies were used, band visualization was performed using Western Lightning® Plus-ECL (PerkinElmer). Signalstain® boost IHC detection reagent (Cell Signaling Technologies), Envision + system, 3′diaminobenzidine-tetrahydrochloride (DAB) (both from DAKO) and anti-rabbit or anti-mouse antibodies were purchased for IHC. To detect mouse vessels a rat specific anti-mouse CD31 antibody (Dianova) was used, with biotinylated anti-rat antibodies, Avidin/Biotin Blocking Kit and VECTASTAIN Elite ABC Kit (all Vector Laboratories) in combination with DAB.

### Evaluation of TKI response

For the *in vivo* evaluation of TKI sensitivity 28 mice [passage 2 (n = 8) and passage 4 (n = 20)] were subcutaneously engrafted in both flanks with UZLX-GIST9 and were used in two subsequent experiments. A detailed overview of treatment groups and schedules can be found in Table 1. Assessment of tumour volume and mouse body weight was normalized for baseline values and performed as previously described [20-23]. After three weeks of treatment mice were sacrificed, and tumour specimens were snap frozen in liquid nitrogen or fixed in 4% buffered formaldehyde for further histological and molecular analysis.

**Table 1:** Description of treatment groups

	**Treatment group**									
	**Control**		**Imatinib**		**Imatinib**		**Sunitinib**		**Regorafenib**	
Passage	N	Dose	N	Dose	N	Dose	N	Dose	N	Dose
2	2	Untreated	3	50 mg/kg/BID	n/a	n/a	3	40 mg/kg/QD	n/a	n/a
4	6	Untreated	4	50 mg/kg/BID	4	100 mg/kg/BID	n/a	n/a	6	30 mg/kg/QD

N = number of mice; n/a = not available.

### Histopathology

Fixed tumour specimens were embedded in paraffin blocks and cut into 4 μm sections on the Microm HM 340 E microtome for haematoxylin and eosin staining (H & E) or IHC. Histological response (HR) was graded by assessing the magnitude of necrosis, fibrosis and myxoid degeneration: grade 1 (0%–10%), grade 2 (>10% and ≤50%), grade 3 (>50% and ≤90%), and grade 4 (>90%) as described before [24,25]. Assessment of mitotic and apoptotic activity was done by counting mitotic figures and apoptotic cells in 10 high power fields (HPF; 400-fold magnification) on H & E staining. Additionally, mitotic and apoptotic activity were assessed by counting respectively pHH3 and Cl-PARP positive cells in 10 HPF. On Ki-67 immunostainings the proliferative activity was scored as the average percentage of stained tumour cells in 5 digital microscopic pictures taken at 400-fold magnification. Microvessel density (MVD) was calculated as the average number of CD31stained vessels in 5 microscopic fields at 200-fold magnification. An Olympus LH-30 M microscope equipped with Color View digital camera was used for all analyses, and Cell D imaging software was utilized for picture analysis (all Olympus).

### Western immunoblotting

For Western immunoblotting, tumour lysates were prepared from snap frozen tumour specimen as described previously [22]. Chemiluminescence levels were captured using FUJI-LAS mini 3000 system (Fujifilm).

### Statistical analysis

Wilcoxon’s matched paired (WMP) test was used to compare tumour volumes on day 0 (baseline) *versus* the last day of the experiment. The comparison between different treatment groups was performed using the Mann–Whitney U (MWU) test. The statistical significance level was defined as p < 0.05 and the STATISTICA software (Stat Soft, version 12.0) was used for all calculations.

## Results

### Histopathological and molecular characteristics

**Figure 1:**
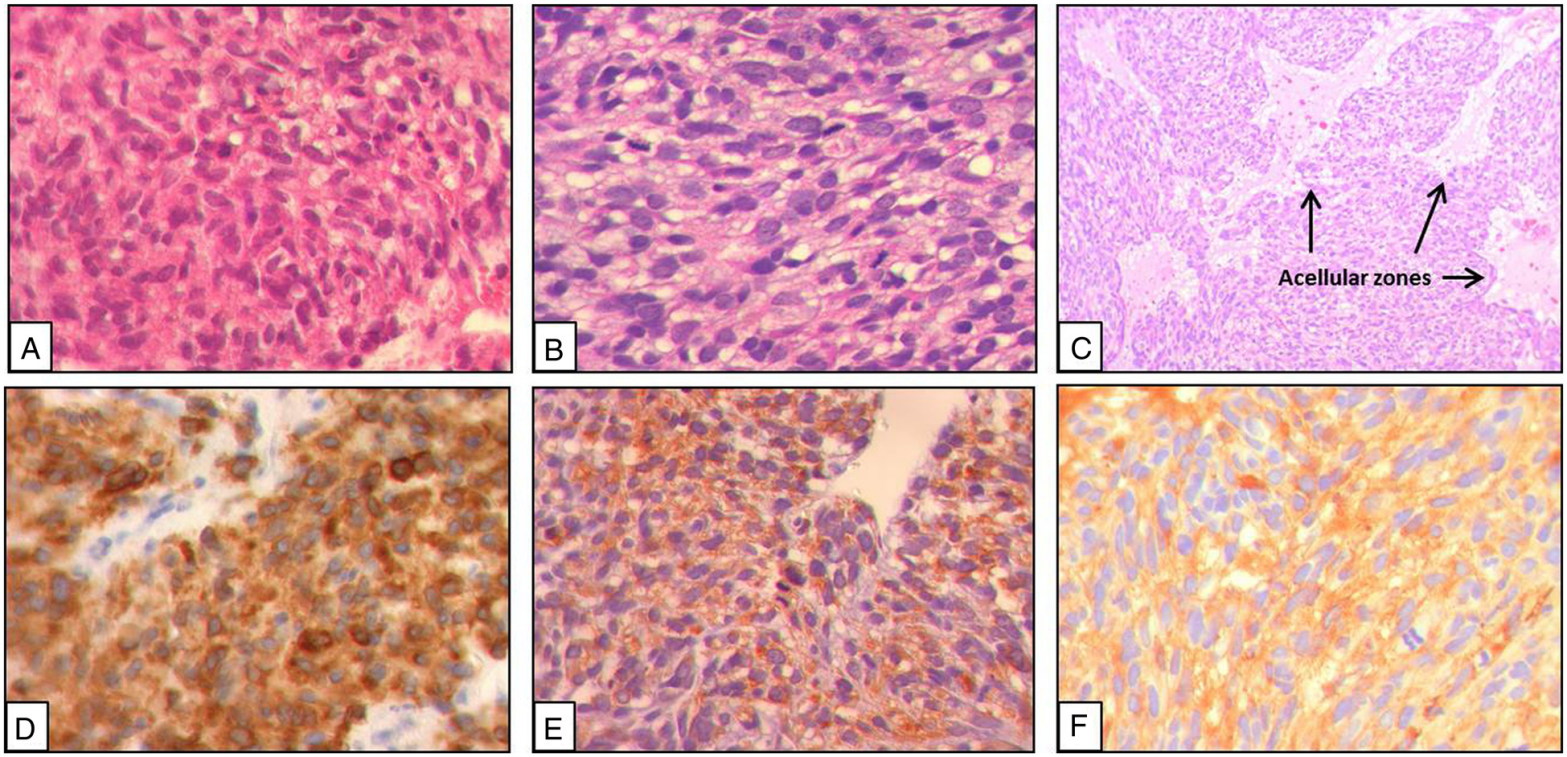
**Haematoxylin and eosin stainings and immunostainings of patient biopsy and UZLX-GIST9 xenograft tumours (passage 4).** H & E stainings at 400X magnification of original intraspinal GIST metastasis **(A)**, and the UZLX-GIST9 xenograft model **(B)**. H & E staining UZLX-GIST9 tumour showing acellular zone **(C)**. CD117 immunostaining of the patient specimen obtained during neurosurgical intervention **(D)** and UZLX-GIST9 xenograft **(E)**. DOG1 staining of UZLX-GIST9 tumours **(F)**.

Since April 2012, the UZLX-GIST9 xenograft model has been maintained by sequential transplantations in NMRI nude mice and currently reached the 7th passage. Histopathological evaluation showed a similar morphological appearance of the ex-mouse UZLX-GIST9 tumours as compared to the original intraspinal GIST metastatic sample obtained from the patient. Microscopic analysis revealed a predominantly spindle cell morphology and diffuse CD117 (KIT) immunoreactivity in the original GIST metastasis and in ex-mouse tumours (Figure 1A, B, D, E), characteristics also observed in the original primary gastric GIST lesion. In addition, the UZLX-GIST9 model displayed strong positivity for DOG1, which is described as a highly sensitive and specific marker for GIST diagnosis (Figure 1F) [26].In general, the histopathological evaluation of UZLX-GIST9 displayed some variability with regards to tumour morphology throughout the tumour sections, revealing tumour areas with high cellular density and more vacuolated regions (Figure 1C). In the latter, a more prominent mitotic activity was observed on both H & E and on pHH3 IHC (data not shown). Of note, acellular zones were commonly observed throughout the tumour area and some apoptotic clustering was observed in surrounding areas (data not shown).

The mutational analysis of UZLX-GIST9 xenografts revealed the same *KIT* exon 11 (p.P577del; W557LfsX5) and exon 17 (p.D820G) mutations as found in the original GIST intraspinal metastasis which was used for engraftment. The mutational profile remained stable throughout the different passages in the mice.

### Xenograft sensitivity to standard TKIs

#### Tumour volume assessment

**Figure 2:**
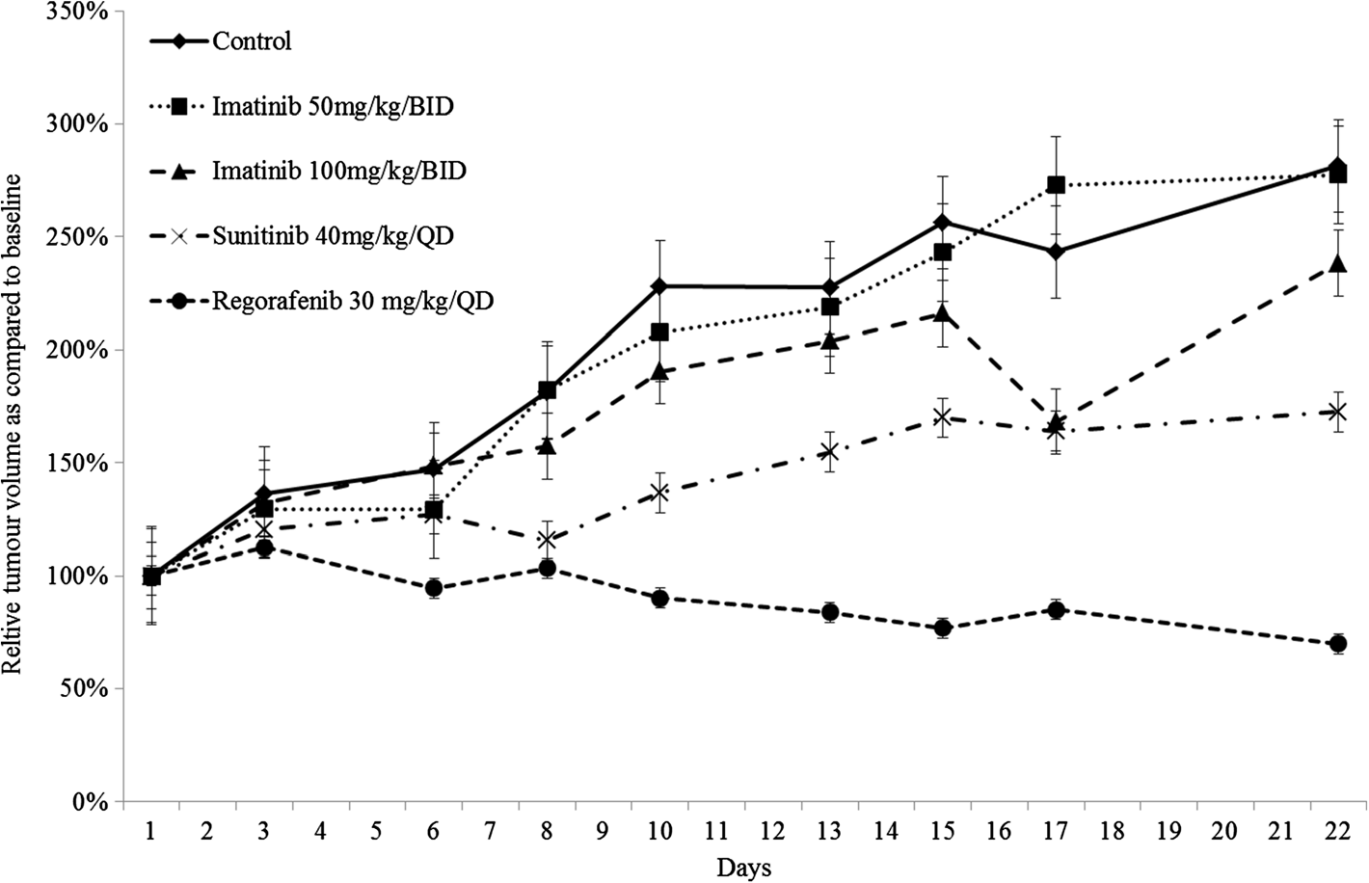
**Relative evolution of tumour volume in UZLX-GIST9 xenografts under treatment with different TKIs.** Data for tumour volume evolution are displayed as means and standard error of the means.

The sensitivity of the UZLX-GIST9 model to standard TKI treatments was evaluated *in vivo.* In general, the tumour burden increased steadily in untreated and imatinib treated cohorts, showing a relative increase to 282, 278, and 238% for untreated, imatinib 50 mg/kg/BID and imatinib 100 mg/kg/BID treated animals as compared to baseline (p < 0.05 for all, WMP test) (Figure 2). No significant difference between relative tumour volumes of untreated and imatinib treated tumour was observed (p > 0.05, MWU test), indicating resistance of the model to the drug. In contrast, sunitinib delayed tumour growth of the xenograft (p = 0.068, WMP test) and regorafenib reduced the tumour volume to 70% of the original size (p < 0.05, WMP test) (Figure 2).

#### Histopathologic evaluation

**Figure 3:**
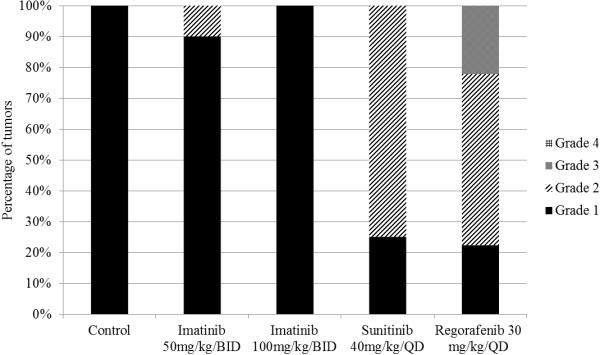
**Assessment of histologic response of UZLX-GIST9 during treatment with different TKIs.** Histologic response was graded by assessing the magnitude of necrosis, myxoid degeneration, and/or fibrosis on H & E staining: grade 1 (0%–10%), grade 2 (>10% and ≤50%), grade 3 (>50% and ≤90%), and grade 4 (>90%) [24,25].

Histologic response (HR) was assessed by evaluating the magnitude of necrosis, fibrosis and myxoid degeneration on H & E stainings of tumours collected at the end of the *in vivo* experiments. HR to TKIs in UZLX-GIST9 was mainly characterized by induction of necrosis. In the imatinib-treated cohorts only minimal histological changes were observed, whereas sunitinib yielded grade 2 HR in 75% of the tumours. Importantly, under regorafenib we observed grade 2 or even 3 HR in almost 80% of tumours (Figure 3).

Untreated UZLX-GIST9 tumours are characterized by brisk mitotic activity (39 mitotic figures/10 HPF on average) on H & E staining. Neither TKI treatment induced a reduction in mitotic activity; rather a slight increase of mitotic activity was observed under TKI treatment, which was even statistically significant in the regorafenib treated cohort (Table 2). No significant induction of apoptotic activity was observed, regardless of the treatment administered (Table 2). Observations on H & E were confirmed by pHH3, Ki-67 and Cl-PARP immunostainings (Table 2).

**Table 2:** Histological assessment of proliferative and apoptotic activity and microvessel density in tumours collected after three weeks of treatment

	**Mitotic and proliferative activity**			**Apoptotic activity**		**Microvessel density**
	**H & E**	**pHH3**	**Ki-67**	**H & E**	**Cl-PARP**	**CD31**
Imatinib 50 mg/kg/BID	↑1.1	=	=	↓1.1	↑1.1	=
Imatinib 100 mg/kg/BID	↑1.1	↑1.1	↑1.1	↓1.3	↑1.3	=
Sunitinib 40 mg/kg/QD	=	↑1.2	↑1.1	↓1.4	↓1.3	↓1.4*
Regorafenib 30 mg/kg/QD	↑1.6*	↑1.5	↑1.1	↑1.1	↑1.4	↓1.4*

Results are shown as fold changes in comparison with control, arrows indicate increase (↑) or decrease (↓). Mann–Whitney U test was performed for statistical assessment; *P < 0.05 (compared to control). H & E - haematoxylin and eosin staining, pHH3 - phospho-histone-H3 immunostaining; Cl-PARP - cleaved PARP immunostaining.

MVD analysis showed that none of the imatinib regiments significantly lowered vessel density as compared to untreated control tumours. In contrast, both sunitinib and regorafenib yielded a 1.4 reduction of microvessel density, which was significant when compared to control (p < 0.05, MWU) (Table 2).

#### Assessment of oncogenic signalling in response to treatment

**Figure 4:**
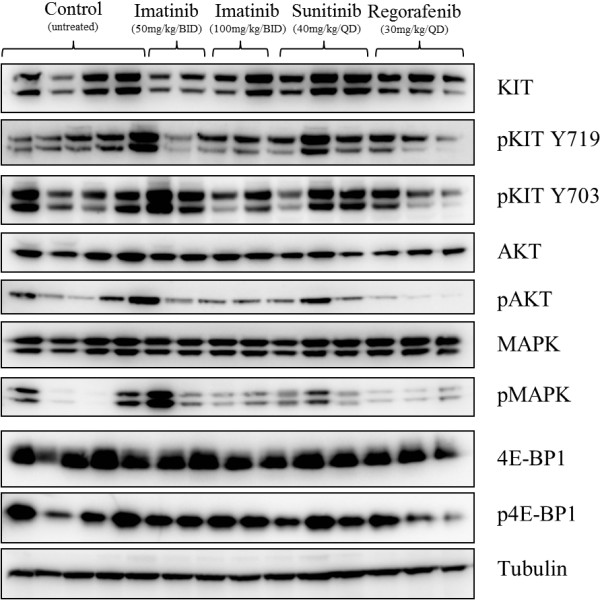
Western analysis of the impact of different TKIs on KIT signalling in UZLX-GIST9.

By Western immunoblotting we observed expression of KIT and its main signalling mediators in all untreated UZLX-GIST9 tumours (Figure 4). In general, imatinib- and sunitinib-treated cohorts did not show a remarkable inhibition of KIT activation and signalling. Under regorafenib, we observed a mild reduction of KIT activation and a slight inhibition of AKT and 4E-BP1 phosphorylation (Figure 4).

## Discussion

Imatinib treatment in GIST is a paradigm for the use of targeted treatments in solid tumours. Nevertheless, despite the remarkable effects of this and other TKIs in GIST, with time the vast majority of patients will inevitably develop resistance against these agents [17]. Importantly, patients progressing under or being intolerant to imatinib, sunitinib and regorafenib are left without any established standard treatment option. Hence, the development of novel therapeutic options is an unmet medical need. Reliable *in vitro* and *in vivo* models are required to test new agents and assess the molecular mechanism of action of innovative drugs.

To date, the number of GIST xenograft models available for research is rather limited. A few xenografts have been established upon subcutaneous injection of GIST-T1, GIST48, GIST430, GIST882 or GIST882Ly cell lines [20,22,27-29]. Of these cell line-derived xenografts GIST48 and GIST430 are described as being resistant to imatinib, although both have shown mild to moderate responses to imatinib in some published *in vivo* experiments [20,27]. Only a few research groups, including our own, have been able to establish GIST xenografts based on patient-derived biopsies [20-23,30-32]. Of these, only the model established by Revheim and colleagues is characterized by a secondary *KIT* exon 17 mutation [30]. The latter model has shown tumour growth under imatinib treatment, although imatinib treatment of this model delayed tumour growth when compared to untreated tumours [33].

Here we are presenting data from a novel GIST xenograft model (UZLX-GIST9) based on a tumour biopsy derived from a patient who had been treated for almost 10 years with a variety of TKIs, including imatinib, sunitinib, regorafenib and nilotinib. To date, the new model is maintained for over two years and 7 passages and continues to grow.

UZLX-GIST9 mimics the morphological, immunohistochemical and molecular features of the original intraspinal GIST metastasis with spindle cell morphology, diffuse CD117 and DOG1 immunoreactivity. The mutational analysis of the xenograft shows *KIT* exon 11 and exon 17 mutations (p.P577del; W557LfsX5; D820G), suggesting resistance to commonly used TKIs.

Indeed, we could show imatinib resistance in the dose range tested. Conversely, the model still showed some level of response to other TKIs (sunitinib and regorafenib), even though we did not observe any anti-mitotic or pro-apoptotic effects of the tested agents. Sunitinib yielded tumour growth delay and regorafenib induced some regression in tumour volume. Most likely, these effects on tumour burden are related to the prominent induction of necrosis observed under regorafenib and sunitinib, which was not seen under imatinib treatment. In addition, only in regorafenib treated tumours a slight inhibition of KIT and AKT activation was observed. The mild increase in mitotic activity in the viable tumour areas under regorafenib treatment is most likely the result of selective pressure by the high degree of necrosis observed in these tumours and the multi-targeted nature of the TKI, resulting in the selection of the most aggressive and mitotically active tumour cells.

These findings provide evidence for the characterization of UZLX-GIST9 as a truly imatinib-resistant model. Intriguingly, despite the lack of a seemingly direct inhibitory effect on cell proliferation and survival we observed a decrease in tumour growth rate or even tumour volume reduction for sunitinib and regorafenib, respectively. Both drugs are known to have anti-angiogenic effects, and we have shown that both drugs significantly reduced MVD in the newly established GIST xenograft model when compared to untreated control tumours [11,15]. We therefore postulate that the effect on tumour volume caused by these drugs could partially result from the antivascular capacity of these drugs; the observation of necrosis as a feature of response supports this hypothesis. Our findings suggest that some of the clinical effects of regorafenib in imatinib-resistant GIST may not be mediated through suppression of KIT signalling. These results warrant further research related to the mechanisms of the regorafenib-induced clinical benefit observed in imatinib- and sunitinib-resistant GIST patients [16]. Additionally, further studies of more potent or more selective anti-angiogenic compounds in the novel UZLX-GIST9 model could prove valuable.

## Conclusions

In summary, a novel human GIST xenograft with *KIT* exon 11 and exon 17 mutations has been established. This novel xenograft model resembles the morphological and molecular features of the original tumour that had failed treatment with four different potent TKIs, including the three approved agents for the therapy of advanced, metastatic GIST. The model exhibits strong imatinib resistance; tumour growth and high tumour cell proliferation was observed under sunitinib. Regorafenib shows significant anti-tumour effects in this model, which seem independent of *KIT* inhibition. Furthermore, both sunitinib and regorafenib significantly lower MVD and induce necrosis in the UZLX-GIST9 model. Overall, we present a novel GIST research tool which will be very valuable for the development of novel experimental treatment approaches and hopefully translate into direct patient benefit.

### Consent

The patient agreed with using a tissue sample collected during routine surgery for research purposes. She could not give written informed consent for the current publication as she died in August 2012.

## Competing interests

The authors declare that they have no competing interests.

## Authors’ contributions

Histomorphology (TVL, YG, RS, GF), molecular analysis (AW, MDR), data collection (TVL, YG, JC, JW, HL, UV, AW), analysis of data (TVL, YG, AW), project coordination (PS, AW), and manuscript preparation (TVL, YG, AW, JC, GF, UV, HL, JW, RS, MDR, PS). All authors read and approved the final manuscript.
